# Ears of the Armadillo: Global Health Research and Neglected Diseases in Texas

**DOI:** 10.1371/journal.pntd.0002021

**Published:** 2013-06-27

**Authors:** Jon Andrus, Maria Elena Bottazzi, Jennifer Chow, Karen A. Goraleski, Susan P. Fisher-Hoch, Jocelyn K. Lambuth, Bruce Y. Lee, Harold S. Margolis, Joseph B. McCormick, Peter Melby, Kristy O. Murray, Rebeca Rico-Hesse, Jesus G. Valenzuela, Peter J. Hotez

**Affiliations:** 1 Pan American Health Organization, World Health Organization, Washington, D.C., United States of America; 2 National School of Tropical Medicine, Baylor College of Medicine, Houston, Texas, United States of America; 3 Sabin Vaccine Institute and Texas Children's Hospital Center for Vaccine Development, Houston, Texas, United States of America; 4 Research!America, Alexandria, Virginia, United States of America; 5 American Society of Tropical Medicine and Hygiene, Deerfield, Illinois, United States of America; 6 University of Texas School of Public Health, Regional Campus, Brownsville, Texas, United States of America; 7 University of Pittsburgh School of Medicine, Pittsburgh, Pennsylvania, United States of America; 8 Dengue Branch, Centers for Disease Control and Prevention, San Juan, Puerto Rico; 9 University of Texas Medical Branch, Galveston, Texas, United States of America; 10 National Institute of Allergy and Infectious Diseases, National Institutes of Health, Rockville, Maryland, United States of America; 11 James A. Baker III Institute for Public Policy, Rice University, Houston, Texas, United States of America

Neglected tropical diseases (NTDs) have been recently identified as significant public health problems in Texas and elsewhere in the American South. A one-day forum on the landscape of research and development and the hidden burden of NTDs in Texas explored the next steps to coordinate advocacy, public health, and research into a cogent health policy framework for the American NTDs. It also highlighted how U.S.-funded global health research can serve to combat these health disparities in the United States, in addition to benefiting communities abroad.

## Introduction

While neglected tropical diseases (NTDs) are usually thought of as a group of chronic parasitic and related infections affecting those living on US$1–2 per day in the poorest developing countries [1], [2], there is increasing awareness that the NTDs also strike pockets of impoverished people who live in wealthy countries, including the United States, Canada, many European nations, and Australia [3]–[9]. The NTDs found among the poor in wealthy countries often differ from those found predominantly in low- and middle-income countries (LIMCs), but they nonetheless exhibit many of the same features, including their chronicity and adverse impact on child development, pregnancy outcome, and worker productivity [4]–[6], [10]. Moreover, the NTDs in the U.S. disproportionately affect people of color and indigenous populations, much as they also do in the Latin American and Caribbean region [3], [11]. In this sense, the NTDs are important contributors to American health disparities.

Today, an estimated 46 million Americans live below the poverty line (defined as US$22,314 for a family of four in the U.S.), including approximately 20 million existing in so-called “extreme poverty” (50% or less than the poverty line) [12], [13]. Some of the largest numbers of people who live below the U.S. poverty line live in Texas [13]–[15]. Roughly one in five Texans (approximately 4–5 million people) currently lives below the poverty line, with South Texas counties exhibiting some of the highest rates of poverty in the U.S. [13]–[15]. In June of 2012, the nonprofit education and advocacy organization Research!America, together with the American Society of Tropical Medicine and Hygiene (ASTMH) and several institutions of the Texas Medical Center including the Sabin Vaccine Institute, Texas Children's Hospital Center for Vaccine Development, and the National School of Tropical Medicine at Baylor College of Medicine, sponsored a one-day forum that explored global health research, social determinants of health, and advocacy to highlight the impact of NTDs in Texas and elsewhere on the Gulf Coast and in the American South. The forum proposed several key steps needed to generate advocacy strategies for the development of evidence-based policies to address these diseases both regionally and nationally.

## Overview of the NTDs in Texas and the American South

The major NTDs in Texas and other areas of the American South are listed in Box 1. Among their common features is the observation that most of these conditions cause chronic disabilities, which disproportionately affect people living in extreme poverty [3], [5], [13]–[15]. Another key feature is that NTDs are important examples of health disparities mostly affecting people of color, particularly African American and Hispanic minorities, largely because of the poverty link [3], [5], [13]–[15].

**Box 1. Major Neglected Tropical Diseases in Texas**

**Parasitic Infections**

- Chagas disease
- Cutaneous leishmaniasis
- Cysticercosis
- Toxocariasis
- Trichomoniasis

**Bacterial and Viral Infections**

- Murine typhus
- Tuberculosis in diabetes mellitus
- Dengue
- West Nile virus

### Neglected Parasitic Infections

**Figure 1:**
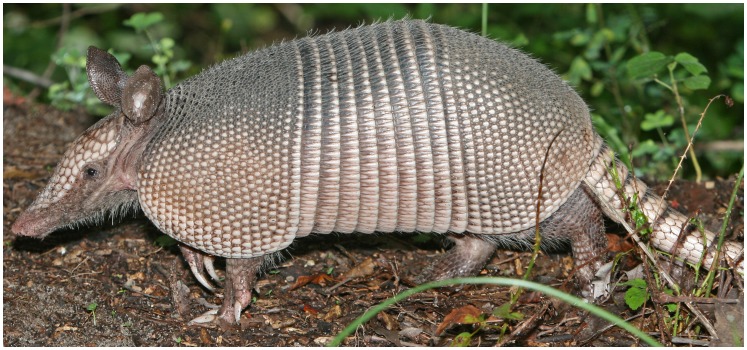
The ears of the armadillo. http://en.wikipedia.org/wiki/File:Nine-banded_Armadillo.jpg, accessed August 16, 2012.

Among the parasitic infections, Chagas disease (American trypanosomiasis caused by *Trypanosoma cruzi* infection) received renewed attention in 2012 based on recently published estimates of large numbers of people infected in the Western Hemisphere, including the high prevalence rates among pregnant women and subsequent maternal-to-child transmission [16], [17]. Of note, the first reported case of mother-to-child transmission in the U.S. was announced on July 6, 2012 by the Centers for Disease Control and Prevention (CDC) [18]. The CDC estimates that 300,000 cases of Chagas disease are found in the U.S. [19], whereas other investigators have suggested that almost as many cases occur in Texas alone [20]. Several kissing bug vector species are widespread in Texas and capable of transmitting *T. cruzi*; a significant percentage of these vectors are polymerase-chain-reaction (PCR) positive for *T. cruzi* [19], [21]. In South Texas, a high percentage of dogs, which are natural hosts, are also infected with *T. cruzi* [22], and a risk map for humans acquiring Chagas disease in Texas has been developed [21]. However, the extent to which *T. cruzi* transmission to humans actually occurs in the state is unknown [14]. There is an urgent need to increase surveillance for human *T. cruzi* infection in the region, possibly through seroprevalence studies, as well as for studies that attempt to document the extent of autochthonous transmission and mother-to-child transmission. In this sense, we are still at the “tip of the iceberg” in terms of our understanding of the epidemiology of Chagas disease in Texas and elsewhere in the American South. An alternative metaphor is that we have only seen the “ears of the armadillo” (similar to the ears of the hippopotamus metaphor sometimes used for malaria in Africa), referring to the nine-banded armadillo (*Dasypus novemcinctus*), which is native to Texas (Figure 1).

Information is also scant for several other key NTDs in Texas. As with Chagas disease, these NTDs appear to be widespread in different areas of the state, but supporting surveillance and transmission studies are either sporadic or missing. Cutaneous leishmaniasis (CL) is another vector-borne parasitic protozoan infection, caused by *Leishmania* spp. and transmitted by sand flies of the genus *Lutzomyia*. Human cases of autochthonous CL caused by *Leishmania mexicana* infection have been recognized in Texas, primarily in the south-central region, since 1903 [23]. In 2008, nine cases were reported in northern Texas, not far from the Dallas-Fort Worth area [24]. There are several important animal reservoirs of *Leishmania* spp. in the Americas. In Latin America, rodents serve as an important animal reservoir for *L. mexicana*, and the Southern Plains woodrat has been implicated in Texas [23] and elsewhere in the southern U.S. Widespread infection of foxhounds in the U.S. with visceralizing *L. infantum* is also of concern, but the true extent of veterinary and human transmission in Texas and the rest of the U.S. is largely unknown [25]. A recent modeling study suggests that the range of reservoirs and sand fly vectors for CL is likely to expand deeper into the U.S., possibly in association with climate change [26], and thus, a northward expansion of CL infection in humans is conceivable.

Among the helminthic infections, neurocysticercosis (NCC) is now a major cause of epilepsy in Texas [27]. Most of the recent cases of NCC are believed to have been imported through immigration from Latin America [27], but autochthonous transmission still remains a possibility. Toxocariasis (*Toxocara canis* and *Toxocara cati* infection) is widespread in the American South, particularly among African American and Hispanic minority populations [28]. A covert form of this NTD has been linked to asthma and developmental delays [29], but the prevalence of toxocariasis in Texas and its potential contribution to chronic sequelae in the state have not been accurately determined.

### Neglected Viral and Bacterial Infections

West Nile virus (WNV) infection, a mosquito-transmitted arbovirus infection, emerged in Houston, Texas in 2002 [30], where it occurs more commonly among people living in proximity to bayous lined with vegetation and other bodies of stagnant or slow-moving water [31]. Texas experienced a historic peak in WNV cases in 2012 affecting several areas of the state [32]. Like other vector-borne NTDs, WNV infection has been linked to poverty and its associated conditions [13], [33], [34]. A study in Houston in 2004 found that 7% of homeless people were positive for WNV infection, and that seroprevalence rose to 17% for those who did not seek shelter at night [34]. Risk factors for severe disease from WNV infection include hypertension, diabetes, and alcohol and substance abuse [35]–[39], which are all chronic morbidities that often go untreated in marginalized populations. In Texas and elsewhere, WNV infection was recently identified as an emerging etiologic agent of chronic renal disease and kidney failure [40].

Dengue emerged in South Texas in 1980, with additional outbreaks recognized in 1999 and 2005, and where conditions related to poverty also represent major risk factors for infection [41], [42]. Studies to determine the prevalence of dengue virus infection among residents of city-pairs on the U.S.-Mexico border have shown much higher rates of acute and past infection in Texas than would have been anticipated based on how infrequently the disease is recognized and reported [41]–[43]. In 2004 and 2005, recently contracted dengue virus infections were found among an estimated 2–4% of the residents of Brownsville, Texas, compared to 7–32% of residents of Matamoros, Mexico, with part of the difference having been ascribed to socioeconomic factors [41], [42]. The under-recognition of an ongoing dengue outbreak in the U.S. was recently highlighted in Key West, Florida [44], [45] and emphasizes the need for better surveillance and education of clinicians about NTDs in the U.S. Severe dengue has occurred in the continental U.S. and is always a concern where frequent dengue virus infections occur. Preliminary studies indicate that dengue may have already emerged in Houston (unpublished data). International air travel increases the risk for importation of dengue virus and possible outbreaks [46], an especially salient factor given Houston's role as a major international air travel hub and the presence of the mosquito vector.

Among the major bacterial infections, murine typhus (*Rickettsia typhi* infection, transmitted by cat fleas) is emerging in South Texas [47]. An important evolving scenario lies at the interface of infectious diseases and the pandemic of chronic disease. Substantial evidence documents type 2 diabetes as the most important major risk factor for tuberculosis (TB) along the Texas border with Mexico [48], [49], increasing the risk of active TB three-fold. Similar observations have now been made in TB high burden countries across the globe [50]. Altered gene expression in the host and altered immune responses to several other pathogens in diabetes have been identified [51]–[54]. Studies conducted in South Texas were instrumental in uncovering the relationship between TB and diabetes such that additional studies in Texas might help in determining if similar relationships exist for other neglected diseases. The interaction between TB and type 2 diabetes illustrates how a neglected disease may interface with a chronic noncommunicable disease (CNCD). Some data suggest that the NTDs themselves manifest much like the CNCDs with respect to their chronic morbidities [55], and may certainly account for a hidden burden of CNCD-related morbidity [56].

## Advancing Public Health in the Region

So far we have only seen the ears of the armadillo with respect to the full extent of the NTD problem in Texas. Tragically, minimal surveillance data exist for all of the NTDs highlighted above [13]. This situation is particularly alarming given that the NTDs disproportionately affect vulnerable populations, especially children, pregnant women, people living in poverty, people of color, and indigenous populations [4]–[8]. To that end, there is an urgent need to identify the critical public health gaps and address the specific needs in Texas and the adjacent regions. Paradoxically, within the U.S. biomedical community there has been a diminished emphasis on population-based investigations to determine disease burden and epidemiology in favor of laboratory and clinical investigations, which are thought to be more amenable to receiving support from the U.S. National Institutes of Health and other funding agencies.

Several types of studies must be undertaken [13]. For many of the major NTDs, there is the need to establish robust estimates of their burden in the U.S. through population-based prevalence and/or incidence studies in Texas and other vulnerable areas, coupled with epidemiologic studies to determine modes of disease transmission. For instance, while we believe Chagas disease is widespread in South Texas, we have only a modest evidence base to support this premise, and we know even less about the percentage of human cases transmitted within the state from indigenous vectors and animal reservoirs [14]. Such information is needed to better understand the dynamics of disease transmission and the potential risk of acquiring this disease. For other NTDs, such as dengue, toxocariasis, and WNV, the factors responsible for urban transmission need to be better delineated. Given that many of these diseases are vector-borne, studies about the prevalence and competence of the relevant hosts and reservoirs are also needed. Collection of this data, which will require work in northern Mexico, may be hindered because of the ongoing unrest in the region. There is a need for better mapping and to conduct geographic information system/remote sensing–based research to produce next generation risk maps for acquiring the NTDs. We need to better understand the exact role of poverty in NTD transmission. Why exactly is poverty a major risk factor for most of the NTDs in the U.S.? Inadequate housing with lack of indoor screens and air-conditioning, as well as external degradation linked with absent sanitation, garbage pick-up, or (in the case of WNV virus) neglected swimming pools [33] may provide part of the answer, but it would be extremely useful to understand the scientific basis for the link between poverty and disease [13]. Some of these factors might be addressed, at least partially, through more outreach by the scientific community to the local citizenry and community-based organizations. For many of the NTDs, case management and treatment algorithms are still at a rudimentary stage. However, once estimates of disease burden are established and populations are identified in the NTD-endemic regions of the U.S., there might be greater interest in conducting treatment and management studies in controlled clinical settings.

Currently, few if any prevention strategies for NTDs are in place in Texas or surrounding states. One exception is an aggressive mosquito-control initiative in Houston and surrounding areas of Harris County to reduce the Culex mosquito population as a means to lower WNV transmission [57]. For all the NTDs, prevention strategies should be defined, the effectiveness of these prevention strategies determined through outcome studies, and their economic and public health value modeled, as has been done for many global health prevention strategies to fight HIV/AIDS, malaria, and other diseases.

It is unclear whether public funds will be available anytime soon to support such public health and prevention studies or to foster community involvement to address the NTD problem in the U.S. There is a dearth of available funding for partnerships with federal (e.g., the CDC), state, and local health agencies for this purpose. One proposal mentioned at the forum is to consider supporting partnering opportunities between major research universities in Texas (e.g., Baylor College of Medicine, University of Texas, Texas A&M University, Texas Tech University, University of Houston, and Rice University) and local universities near the Mexican border (e.g., University of Texas-Pan American) to build local capacity for epidemiologic research. This approach is similar to a U.S. National Institutes of Health Fogarty International Center–sponsored “twinning” initiative in Africa known as MEPI (Medical Education Partnership Initiative) [58]. Possibly, such an approach could become an initiative of The Academy of Medicine, Engineering, and Science of Texas (TAMEST).

Simultaneously, there is a substantial need for workforce development and capacity building within the state to enhance laboratory and diagnostic testing for the NTDs. Many of the current diagnostic assays for conditions such as Chagas disease, cysticercosis, toxocariasis, and the arboviral infections, among others, are not widely or easily available, especially in the impoverished areas of South Texas where the needs are the greatest. In some cases, especially for the neglected viral infections, special containment facilities may be required. Capacity building is needed to train public health officials for the unique needs of surveillance and control of NTDs, including the use of appropriate technology for these conditions. Such efforts include the need to train entomologists or vector biologists in the identification and control of the suspected vector. Physicians, nurse practitioners, and other health care providers must be trained to recognize, manage, and treat these diseases. The new National School of Tropical Medicine in Houston is potentially positioned to work in close partnership with state and local health agencies in this regard [13], [59]. The existing network of University of Texas public health schools and other public health institutions such as the Pan American Health Organization (PAHO) could also enhance training and other collaborations. Previously, PAHO, together with a network of public health laboratories, was highly effective in coordinating efforts to eliminate polio, measles, rubella, and other infections in the Latin American and Caribbean region [60].

## Advancing Research and Development (R&D) for New Control Tools

In tandem with expanded public health measures, there is a pressing need to develop better control tools for the NTDs, including new drugs, vaccines, diagnostics, insecticides, and mathematical and computational models. For example, for Chagas disease, there is an urgent need for new biomarkers to follow disease progression [61]. The toxicities and other problems inherent in the only two existing medicines currently used to treat Chagas disease (benznidazole and nifurtimox) have been highlighted, prompting a search for alternative drugs and new vaccines [17], [62]–[64]. We also need new tools to assess vector exposure and transmission of Chagas disease and leishmaniasis. A vaccine for leishmaniasis (including a transmission blocking vaccine from dogs) has also been proposed [65] and shown to be cost-effective for VL [66]. For NCC, there is a need for more sensitive diagnostic tests and biomarkers, especially for patients with single brain lesions, which have a high false-negative rate [27]. For WNV infection, there is a need to better delineate the role of the virus as a cause of renal disease and to evaluate possible antiviral therapeutic interventions and possibly a vaccine [40], [67]. For dengue, several prototype vaccines are under development by a number of pharmaceutical companies, as well as new antiviral drugs [68]. Of interest is the development of a new humanized mouse model for dengue, which may accelerate such product development [69]. For all of the vector-borne NTDs, at-risk populations would benefit from improved vector and reservoir control strategies and integrated management. Mosquito control methods could benefit prevention efforts for multiple NTDs in Texas, including dengue, WNV, and Saint Louis encephalitis. We recognize the complexities of implementing vector and reservoir control measures and their integrated management; however, each of these control tools can be modeled to determine when they may be cost-effective or highly cost-effective. Some may even prove to be cost-saving (economically dominant), when an intervention actually saves money (in addition to having health benefits) compared to the status quo. Many of these products may not be financially remunerative and would need to be developed in the nonprofit sector, possibly through similar models as those used by product development partnerships [70]. Finally, and as pointed out above, the finding of syndemic tuberculosis and type 2 diabetes in Texas should also prompt the search for additional links between NTDs and CNCDs. Such research could play an important role in improving health interventions for these conditions.

## Strategies for Advocacy: The Way Forward

Given the overall dearth of information currently available on the NTDs in Texas and the rest of the U.S., it is challenging to formulate useful policy guidelines that are fully evidence-based. Yet, without a coordinated plan of advocacy and education on these conditions, public health–directed research and development efforts will not increase, leaving us in the same situation of insufficient data needed to make informed policy decisions, and insufficient interventions to address known threats. It is also true that formulating a successful advocacy strategy faces an uphill battle. NTDs have already fallen through the cracks between the two large international public health movements inaugurated in this first part of the twenty-first century: twelve years of global health outreach beginning in 2000 with the launch of the Millennium Development Goals and the more recent CNCD advocacy effort launched at the United Nations last year. Domestically, while there has been increased attention to health disparities in the U.S. during the landmark health care reform measures over the past two years, the American NTDs, an important contributor to this disparity, have been barely mentioned. To that point, the first summit on NTDs in the U.S. held in Washington, D.C. more than two years ago [6] helped to stimulate the introduction of the “Neglected infections of impoverished Americans act of 2011” (H.R. 528) as a means to encourage U.S. health officials to collect essential information on some of the major diseases highlighted above [13], [71]. However, it is unclear whether this effort will be implemented.

There are several possible reasons why the NTDs in the U.S. have not risen higher on the domestic or global health policy agenda, including the fact that these diseases often have complicated names, ecologies, and modes of transmission. It is also likely that NTDs face the same funding obstacles as other diseases that disproportionately affect people of color and those living in extreme poverty. During the Texas forum, it was noted that if these conditions were affecting people living in wealthy suburbs, they might have already gained substantial media attention or even possibly become the subject of congressional hearings. Instead, the U.S. NTDs remain “forgotten diseases of forgotten people” [72]. In this sense, the American NTDs put to the test the nation's commitment to a core principle of public health: an issue of equitable benefits and justice for all populations. But beyond the moral implications, because these diseases respect no border or neighborhood boundaries, even skeptics should understand that tackling diseases of poverty now will confer the pragmatic benefit of dramatically reducing the risk of spread.

Simply put, there is an urgent need to bolster the evidence base for defining the scope and impact of the NTDs in Texas and surrounding states. In Latin America, careful disease burden assessments and cost-effectiveness studies of vaccination and other intervention strategies helped to introduce new vaccines against key diseases [73]. Many of these activities are coordinated through PAHO's ProVac Initiative [73]. Ideally, funds would be made available to launch a similar initiative for NTDs in the U.S., but instead we are faced with a “chicken and egg” situation. We don't have the necessary funding, political will, or infrastructure to support data collection for surveillance, transmission, and disease burden studies, much less the investment needed to produce new interventions. Given these realities, the need for a comprehensive advocacy strategy that factors in these innovative strategies is essential if we are to make a real difference in the lives of “forgotten people” in Texas and elsewhere. However, without more public health evidence and examples of the benefits of new tools, effective advocacy strategies are extremely difficult to formulate.

Despite the hurdles, we must persevere. Research!America, ASTMH, and some of the key Texas universities and institutions can take meaningful first steps by embarking on media outreach, together with science-policy discussions in Washington, D.C., and by engaging the Texas delegation of the U.S. Congress and key congressional caucuses (e.g., the Black and Hispanic Caucuses and the Malaria-NTD Caucus). It is important that we take every opportunity to obtain the data required to make informed decisions and engage in targeted advocacy. Where appropriate, the CDC and other research-oriented institutions or organizations should present their latest epidemiologic and disease burden data offering an informed perspective on both the domestic and international front. With a coordinated effort of multiple stakeholders both within and outside of U.S. government, we can overcome ignorance and apathy and combat NTDs as a compelling and urgent health disparities issue, an emblematic public health imperative, and an insidious global health threat.
